# The Role of Circadian Rhythm Dysregulation, Abnormal Sleep Patterns, and Sleep Disorders on the Development of Diabetes

**DOI:** 10.3390/clockssleep8020022

**Published:** 2026-04-28

**Authors:** Hulya Merie, Bashair M. Mussa, Salah Abusnana

**Affiliations:** 1 Basic Medical Science Department, College of Medicine, University of Sharjah, Sharjah P.O. Box 27272, United Arab Emirates; u22105091@sharjah.ac.ae; 2 Clinical Science Department, College of Medicine, University of Sharjah, Sharjah P.O. Box 27272, United Arab Emirates; sabusnana@sharjah.ac.ae; 3 Diabetes and Endocrinology Department, University Hospital Sharjah, Sharjah P.O. Box 27272, United Arab Emirates

**Keywords:** diabetes, circadian rhythm, sleep duration, sleep disorders, sleep quality, chronotype

## Abstract

It is noteworthy that disturbances in circadian rhythms and irregular sleep patterns can exert influence over the onset of Type 2 Diabetes (T2DM). Similarly, they can impact the development of Type 1 Diabetes (T1DM). In recent decades, there has been a notable trend towards both reduced and extended sleep durations, with a concurrent rise in occurrences of compromised sleep quality attributable to sleep fragmentation. These sleep disturbances, along with clinically recognized sleep disorders such as sleep apnea and insomnia, have been increasingly associated with a range of detrimental health outcomes. Of particular concern is the growing evidence linking sleep dysregulation to an augmented risk of metabolic diseases, including diabetes. In addition to sleep duration and quality, emerging research suggests that an individual’s chronotype, reflecting their preferred time for going to sleep, may also exert an influence on disease development, particularly T2DM. The habit of going to bed late when compared to the tendency of going to bed early tends to cause significant disruptions to daily social engagements. Eventually, this misalignment may lead to discrepancies in sleep schedules between weekdays and weekends, commonly referred to as social jetlag. The current review aims to discuss the complex relationship between circadian rhythm misalignment, triggered by improper sleep habits such as short or long sleep duration, disrupted chronotype, social jetlag, and sleep disorders, on the subsequent impact on the development of diabetes. Overall, current evidence suggests that circadian rhythm disruption and sleep disorders contribute significantly to metabolic dysregulation and diabetes risk, highlighting the importance of sleep health in prevention and management of diabetes.

## 1. Introduction

Diabetes mellitus is a group of metabolic disorders characterized by chronic hyperglycemia resulting from defects in insulin secretion, insulin action, or both. The most common forms include type 1 diabetes mellitus (T1DM), which results from autoimmune destruction of pancreatic β-cells, and type 2 diabetes mellitus (T2DM), which is primarily associated with insulin resistance and progressive β-cell dysfunction. T2DM affects approximately 90% of individuals diagnosed with diabetes, while T1DM constitutes the majority of the remaining 10% [1]. Despite their distinct pathophysiological mechanisms, both T2DM and T1DM are characterized by anomalous glucose metabolism leading to potential vascular complications and premature mortality [2]. The impact of diabetes on an individual’s quality of life is significant, as it necessitates continuous glucose monitoring and control, which can also contribute to depression and psychological issues. The prevalence of diabetes has dramatically increased over the past few decades, mostly because of the persistent rise in the incidence of T2DM. In 2024, more than 530 million adults were suffering from the disease worldwide, and it is anticipated that this number will keep growing [3]. Among the factors attributing to this dramatic spike in diabetes are sedentary lifestyles, obesity, and the excessive intake of unhealthy food [4]. Evidence suggests that impairment of the circadian rhythm due to irregular sleep patterns is also considered among the risk factors contributing to diabetes [5].

Due to the development of industry and the widespread use of mobile devices, television, and the internet, people may spend more time indoors these days without observing the natural cycles of lighting. Because everything is always available in our developed world, more individuals have started seeing sleep as unnecessary and must be avoided to meet social and professional obligations [6]. This alteration in sleep–wake behavior has increased the rate of disruption in society’s sleep cycle and circadian rhythm. Furthermore, the development and management of diabetes appear to be affected by the quality and amount of sleep each day [7]. Less sleeping hours, irregular sleep patterns, and common sleep disorders of insomnia and obstructive sleep apnea (OSA) are all linked to a greater risk of prevalence of diabetes and may indicate poorer outcomes in people who already have the condition [8]. It is an unexpected finding that not only does short sleep duration affect the risk of diabetes, but also long sleep durations were found to have a significant influence on the development of T2DM, denying the possibility of a straightforward linear relationship between hours of sleep and metabolic illness [9]. Supporting these findings, it was revealed that patients with OSA who experience shorter and more fragmented sleep cycles had a higher risk of T2DM even after adjusting for other risk factors like age and obesity that are associated with both OSA and T2DM [10,11].

Knowing that a possible association between the disruption of the wake–sleep cycle and diabetes exists, it is crucial to investigate the potential role of the circadian rhythm and sleep in blood glucose control and the development of diabetes. As a result, this review aims to offer a comprehensive outline of the underlying mechanisms through which sleep dysregulation and sleep disorders contribute to the development of T2DM. It aims to provide an extensive analysis of the available evidence elucidating how circadian misalignment and sleep disruption encompassing factors such as sleep duration, chronotype, and social jetlag, as well as fragmented sleep resulting from sleep disorders, can potentially lead to the onset of T2DM. Additionally, it seeks to offer a general overview of the relationship between circadian misalignment/sleep disruption and T1DM.

### Exploring the Complexities of Sleep and Circadian Rhythm

It is important to understand the proper physiology of sleep to understand how the circadian system gets disrupted. Sleep is a needed natural process in humans that is controlled by circadian rhythms [12]. The circadian system was developed to produce cyclical patterns in biological and physiological processes. These rhythms are synchronized with the 24 h environmental cycles caused by the rotation of the Earth [13]. The daily rhythms experienced by the human body every day are caused by the interplay between endogenous 24 h rhythms that persist under stable conditions, and behavioral and environmental factors [14]. The central master clock, found in the suprachiasmatic nucleus (SCN) region of the hypothalamus, together with the peripheral clock found in almost every organ including the liver, muscle, and adipose tissue, generates a complex network of oscillations that create the endogenous circadian rhythm [15]. The circadian expression of certain genes involved in a range of physiological activities is regulated by the peripheral clocks, which perform a crucial and distinctive role in each of their relative tissues [16]. SCN, which are made up of several circadian oscillators, are situated in the anterior hypothalamic region of the brain and emit coordinated circadian signals [17]. The clock genes including *CLOCK*, *BMAL1*, *PER*, and *CRY*, which are responsible for the 24 h cycles of gene transcription, are among the immediate early genes in the SCN that react to light signals. The same clock genes produce delayed, interlocked negative feedback loops in gene transcription and translation in cells throughout all physiological tissues [18].

In this review, several key terms related to sleep and circadian biology are used. Sleep dysregulation refers to irregular sleep timing or duration that disrupts normal physiological processes and circadian alignment. Circadian disruption describes the misalignment between the internal biological clock and external environmental cues, such as the light–dark cycle or social schedules. Sleep deprivation refers to insufficient sleep duration relative to physiological requirements. In addition, sleep loss can influence appetite-regulating hormones, including leptin, ghrelin, and insulin, which are often referred to as feeding hormones because of their role in regulating energy balance, appetite, and glucose metabolism. In this review, the term sleep disorders primarily refer to conditions such as insomnia, OSA, periodic limb movement disorder, and central sleep apnea, which have been increasingly associated with metabolic dysregulation and diabetes risk [19,20,21,22].

Although light serves as the primary stimulus for the SCN, it was revealed that the circadian cycle, which includes the sleep–wake cycle, lasts longer than 24 h in blind people [23]. This discovery gave rise to the hypothesis that the human biological clock may be stimulated by sources other than light. Thermal, hormonal, and neural pathways, as well as nutrition, physical activity, and the feeding/fasting state, were found to be among the effective circadian stimuli in multiple peripheral organs [16]. Disruption of sleep and circadian rhythms contribute to metabolic disturbances, particularly insulin resistance and diabetes. Consequently, the disruption of sleep and circadian rhythms has been implicated in the potential development of metabolic disorders. The subsequent sections will delve further into the intricate relationship between sleep-circadian dysregulation and the onset of T2DM, shedding light on the underlying mechanisms and supporting evidence. While less evidence is available regarding the association between circadian rhythm dysregulation and T1DM, the final section will briefly explore this relationship with T1DM.

## 2. Type 2 Diabetes

### 2.1. Mechanisms Linking Sleep Duration and T2DM

Regarding short sleeping durations, several studies have looked at the possible mechanisms by which sleep deprivation may adversely influence the body’s metabolism by altering energy metabolism, pancreatic β-cell function, and insulin sensitivity. The first experimental study conducted by Spiegel et al. observed that 11 healthy men experienced increased levels of ghrelin by 28% and reduced levels of leptin by 18% after two nights of 4 versus 10 h of sleep. Increased feelings of hunger and appetite ratings of 23% and 33% were reported, respectively. Notably, the unbalanced feelings of an increased desire to eat were reduced by extending the duration of sleep [24]. The results of a subsequent systematic study also supported this finding [25]. This study’s comprehensive review also revealed a link between less sleep and higher caloric consumption by an average of 259 kcal per day. The increased appetite appeared to be specifically connected to more carbohydrate intake. This reported increase in appetite was shown to be mostly for sweets, desserts, and carbohydrates. Multiple studies have also examined how sleep deprivation affects energy expenditure. Patterson et al.’s study suggested individuals who started getting 9 h of sleep on average expended 113 fewer calories per day in physical exercise than those who got less than 6 h of sleep per day [26]. On the other hand, resting energy expenditure is not thought to be enhanced by sleep deprivation, yet physical activity appears to become lower with sleep deprivation [27].

Numerous factors, including hormonal changes that control energy balance, contribute to the relationship between insufficient sleep and excessive energy intake. Additionally, those who get less sleep have more available time while they are non-asleep, and this can likely result in more caloric intake. The effect of sleep deprivation on brain activity in response to food cues is another significant relationship between decreased sleep and increased food intake. Reward-related brain networks are more activated when sleep is restricted [22]. This alteration may promote increased energy intake, weight gain and metabolic dysregulation, thereby contributing to the development of insulin resistance and T2DM [28,29].

Furthermore, another mechanism underlying sleep deprivation and its effect on the development of diabetes was observed by Broussard et al. [30]. She reported that sleep deprivation was linked to a decline in subcutaneous adipose cell insulin sensitivity. There are several mediating mechanisms that might have a role in this association. Restricted sleep is linked to the activation of the sympathetic nervous system (SNS) which possesses actions that both stimulate insulin resistance and hinder insulin secretion. A rise in sympathetic activity also validates the amount of free fatty acids (FFAs) in the plasma due to lipolysis. Consequently, this may cause ectopic fat deposits around the liver and muscles, resulting in insulin resistance. Insufficient sleep has also been associated with higher levels of plasma cortisol, as well as a lack of the natural drop in cortisol at the end of the day [31].

Sleep restriction has also been shown to influence neural pathways involved in food reward and appetite regulation. Experimental studies suggest that insufficient sleep increases activation of brain regions associated with reward processing, leading to greater responsiveness to high-calorie food cues. These alterations may promote increased energy intake, weight gain, and metabolic dysregulation, thereby contributing to the development of insulin resistance and T2DM [21].

In addition to short sleep duration, long sleep duration has also been associated with an increased risk of T2DM, forming a U-shaped relationship. The underlying mechanisms are less clearly understood but may involve low-grade inflammation, reduced physical activity, underlying comorbidities, and circadian misalignment. Long sleep duration may also reflect poor sleep quality or fragmented sleep, which can negatively affect glucose metabolism and insulin sensitivity [32].

### 2.2. Mechanisms Underlying Sleep Disorders and the Development of T2DM

Through a few different mechanisms, sleep disorders may contribute to insulin resistance and β-cell malfunction [33]. These mechanisms include SNS activation, systemic inflammation, oxidative stress, and hormonal dysregulation, all of which may impair insulin signaling and pancreatic β-cell function. In people with sleep disorders, hypoxia, sleep fragmentation, and SNS activation are some of the mechanisms that significantly contribute to the development of T2DM [1,10,34]. Increased sympathetic responses and greater inflammation are the outcomes of fragmented sleep [35]. Moreover, sleep fragmentation not only has the potential to contribute to obesity but may also induce adipose tissue inflammation mediated by NADPH oxidase-2 [36]. It has been found that intermittent hypoxia and sleep deprivation, which are prominent features of OSA, are critical factors in the impaired glucose metabolism observed in this condition, working synergistically with obesity [37]. Therefore, OSA contributes to glucose intolerance, ultimately leading to T2DM [38].

Sleep disorders disrupt the balance between the parasympathetic and sympathetic nervous systems during sleep. Frequent sleep disturbances elevate sympathetic tone, leading to increased load on the circulatory system, basal metabolism, stress hormone levels, and a higher risk of developing insulin resistance or diabetes [39]. Research findings point to the relationship between sleep disorders and diabetes as bidirectional. Sleep disorders can contribute to the development or worsening of diabetes, while diabetes itself, particularly with poor metabolic control, often leads to sleep disorders [5]. In addition to that, lower melatonin secretion was found to be independently associated with a higher risk of developing T2DM [40].

**Figure 1:**
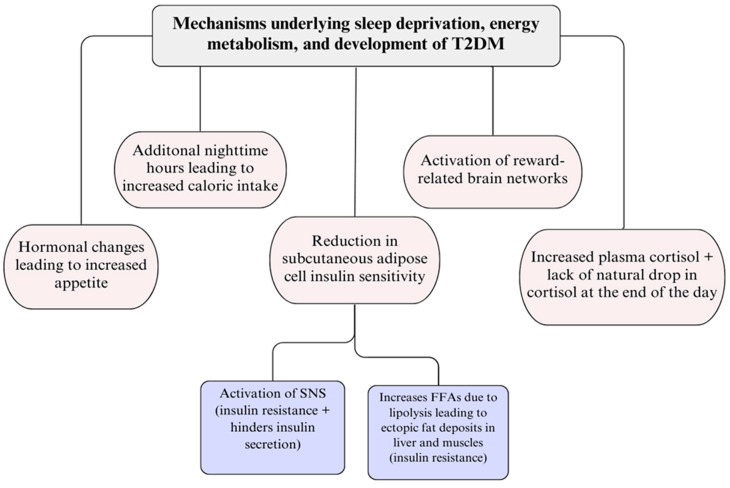
Mechanisms Linking Sleep Restriction and Fragmentation to Metabolic Dysregulation and the Development of Type 2 Diabetes.

Hypoxia, particularly intermittent hypoxia in sleep apnea, is another underlying pathway that contributes to insulin resistance through mechanisms such as increased production of reactive oxygen species (ROS) and inflammation of adipose tissue [41,42]. Notably, chronic intermittent hypoxia has been proposed as the most crucial factor in the development of insulin resistance among individuals with sleep apnea [43]. Intermittent hypoxia may directly impede the functioning of β-cells and impact liver enzymes and liver function, thereby affecting glucose regulation. Moreover, chronic intermittent hypoxia may influence skeletal muscles and their ability to uptake glucose [44]. Experimental studies suggest that insufficient sleep increases activation of brain regions associated with reward processing, leading to greater responsiveness to high-calorie food cues. These alterations may promote increased energy intake, weight gain, and metabolic dysregulation, thereby contributing to the development of insulin resistance and T2DM. These pathways and diabetes-related complications contributing to sleep disturbances are summarized in Figure 1 [28,29].

In individuals with long-standing diabetes, several diabetes-related complications may further contribute to sleep disturbances. Diabetic autonomic neuropathy can disrupt normal cardiovascular and respiratory regulation during sleep, increasing the risk of both obstructive and central sleep apnea. Peripheral neuropathy may also cause nocturnal pain and discomfort, which can lead to frequent awakenings and poor sleep quality. Furthermore, periodic limb movements during sleep have been reported more frequently in individuals with diabetes and may contribute to sleep fragmentation. In addition, diabetic nephropathy and cardiovascular complications may further impair sleep quality through mechanisms involving inflammation, fluid redistribution, and metabolic dysregulation [45].

### 2.3. Mechanisms Linking Circadian Rhythm Disruption and T2DM

Circadian rhythm disruption plays a critical role in the development of T2DM through several biological pathways. The circadian system regulates glucose metabolism, insulin secretion, and energy balance through central and peripheral clocks. Misalignment between the internal circadian clock and external behaviors, such as irregular sleep patterns or shift work, can impair glucose tolerance and insulin sensitivity [46].

At the molecular level, circadian clock genes regulate pancreatic β-cell function and hepatic glucose production. Disruption of these genes has been associated with impaired insulin secretion and increased metabolic risk. Hormonal factors also play a key role. Melatonin, a hormone regulating circadian rhythm, influences insulin secretion and glucose homeostasis [13]. Reduced melatonin levels or altered secretion timing have been associated with increased risk of T2DM. Additionally, circadian misalignment may increase cortisol levels, sympathetic activity, and inflammation, all of which contribute to insulin resistance [47].

## 3. Circadian and Sleep-Related Factors Contributing to the Development of T2DM

### 3.1. Chronotype and Social Jetlag

In our developed nations, individuals have artificial light that is available continuously, and this may impact their bedtime and activity choices, this is known as a chronotype [48]. In other words, the term “chronotype” describes a person’s preferred circadian rhythm, and it can be roughly categorized as “morningness” or “eveningness”. People with the morning chronotype prefer going to bed early and tend to complete their tasks in the early morning, whereas evening chronotypes favor staying up late at night [49]. Some individuals who prefer the evening chronotype may experience a persistent form of sleep misalignment which is referred to as “social jetlag”. Due to their late sleep onset and early arousal, they may experience significant sleep debt during the workweek, which they must make up for by sleeping longer on work-free days. The behavioral rhythm is therefore out of sync with their endogenous circadian clock. Identifying this social jetlag is performed by measuring the discrepancy in mid-sleep time between busy days and work-free days [50]. Chronotype refers to an individual’s natural preference for sleep and activity timing within the 24 h day, often categorized as morning, intermediate, or evening types. SJL refers to the mismatch between an individual’s internal circadian clock and externally imposed social schedules, such as work or school timing, which can lead to chronic circadian misalignment [51].

Considering sleep misalignment and evening chronotype, it is expected that metabolic diseases and adverse health issues may arise. A controlled experimental study demonstrated that circadian disruption led to hyperglycemia, insulin resistance, and systemic inflammation among individuals without preexisting illnesses [52]. Epidemiological data further supported the notion that individuals with an evening chronotype and shorter sleep duration were more likely to engage in undesirable habits, including physical inactivity and poor dietary choices [53]. Additionally, despite a longitudinal study showing a greater rate of T2DM in adults with evening chronotypes [54], research on social jetlag showed varying findings. Specifically, SJL was found to be significantly linked to a higher risk of metabolic syndrome and diabetes/prediabetes in participants under the age of 61 [55]. Conversely, a separate cohort study revealed that SJL was related to obesity in early chronotypes only [56]. While some studies have indicated a correlation between late evening chronotype and poor glycemic control in patients with diabetes [57,58], another study showed no relationship between SJL and HbA1c levels in patients with T2DM [59]. In addition to chronotype and SJL, shift work is an important contributor to circadian disruption. Several studies have shown that individuals engaged in night shift work have a higher risk of developing T2DM. This increased risk is likely due to chronic circadian misalignment, irregular sleep patterns, and associated lifestyle factors [60].

### 3.2. Sleep Duration

Extensive research has demonstrated a consistent association between shorter and longer sleep durations and an increased risk of diabetes. For instance, conducted a study in which males with prediabetes were followed for seventeen years, and their sleep duration was categorized into five groups (5, 6, 7, 8, and >8 h) [59]. The analysis revealed that individuals who slept ≤5 or 6 h had twice the risk of developing diabetes, while those who slept >8 h had three times the risk [61]. Similarly, a study conducted on Taiwanese adults showed that those who have ≤5 h of sleep had nearly a 2 times higher risk for diabetes than those who have 7–8.9 h of sleep each day. This study specifically highlighted the strong correlation between shorter sleep duration and a higher prevalence of diabetes, particularly among young adults [62].

Cross-sectional studies have consistently demonstrated a U-shaped relationship between sleep hours and the risk of diabetes [63,64]. Compared to individuals who reported regular sleep hours (7 h per day), those with short sleep duration (5–6 h per day) have a two-fold increased risk of prediabetes and T2DM. Conversely, individuals with long sleep duration have a nearly 60% increased risk of developing T2DM. A meta-analysis further supported these findings, showing that the lowest risk for T2DM was associated with 7–8 h of sleep per day, while both short and long sleep durations were linked to an increased risk for T2DM [19]. Furthermore, it was observed that males were more susceptible than females to the association between shorter sleep duration and the incidence of T2DM [65,66]. This finding was further confirmed by a recent cohort study where individuals were followed up for 16 years, those who slept for 5 h or less were more susceptible to T2DM, particularly among males [67]. A comprehensive meta-analysis emphasizing risk factors for T2DM development highlighted the significance of sleep disturbances, such as insomnia, OSA, and sleep apnea. Interestingly, this meta-analysis revealed that sleep disturbances had a greater impact on the risk of diabetes compared to physical inactivity, with obesity and family history still having the greatest influence on T2DM risk [68].

A recent meta-analysis showed that the incidence of T2DM was 4.73% in individuals with short sleep duration (≤6 h), 4.39% in those with normal sleep duration (6–9 h), and 4.99% in individuals with long sleep duration (≥9 h). This meta-analysis indicates that both short and long sleep durations increased the risk of T2DM compared to normal sleep duration [69]. However, it is important to note that two other meta-analyses reported no significant association between shorter and longer sleep hours and the risk for diabetes [70,71]. This may be explained by the lack of published research on the desired result, which does not always imply the absence of a substantial association. Furthermore, a study by [70] focused on 51 Emirati patients diagnosed with T2DM as illustrated in Table 1, shedding new light on the potential benefits of a personalized approach to enhancing sleep quality, which involves tailoring interventions based on an individual’s sleep patterns, circadian rhythm profile, lifestyle behaviors, and metabolic risk factors to improve sleep quality and glycemic control [72]. This study represented the first of its kind to investigate the effects of targeted interventions aimed at improving sleep patterns in this specific population. The findings revealed a notable positive impact on the glycemic control of obese patients with T2DM [73].

### 3.3. Sleep Disorders and T2DM

Having sleep disturbance is a major risk factor for developing T2DM, although it is often overlooked. Both glucose metabolism and weight control are significantly impacted by poor sleep quality and short sleep duration resulting from sleep disorders [68]. Having a sleep duration of 5 h and an unfavorable sleep quality are both risk factors for developing T2DM; similarly, insomnia and OSA are also risk factors for developing T2DM. Hence, it is possible that controlling and resolving sleep disturbances may be crucial in preventing T2DM. Given the link between sleep disorders and the onset of T2DM, patients with T2DM are more likely to experience sleep disturbances than the healthy population.

Sleep disorders represent a heterogeneous group of conditions that may affect metabolic health. In addition to commonly studied disorders such as insomnia and OSA, other sleep disorders including central sleep apnea, periodic limb movement disorder, and disorders of hypersomnolence have also been associated with metabolic disturbances. These disorders may influence glucose metabolism through mechanisms such as sleep fragmentation, intermittent hypoxia, inflammation, and autonomic nervous system dysregulation. Patients with narcolepsy, characterized by orexin deficiency, exhibit increased metabolic risks, including insulin resistance and T2DM [74,75,76].

#### 3.3.1. Insomnia

One of the most prevalent sleep disorders is insomnia. Insomnia is characterized by difficulty initiating sleep, difficulty maintaining sleep, or early morning awakening despite adequate opportunity and appropriate conditions for sleep [23].

A recent meta-analysis with a total of six studies revealing the impact of insomnia disorder on the development of diabetes showed that insomnia disorder with short sleep duration (<6 h) was associated with a higher risk of T2DM (RR = 1.63) [76]. Similarly, a meta-analysis comprising 71 papers revealed that [77], in patients with T2DM, the frequency of insomnia and its symptoms is 4 folds greater than in the healthy population. The prevalence increased more with greater age (44%) or with the presence of comorbidities (60%). In addition, the data showed links between insomnia and adverse health outcomes including higher HbA1c (mean difference, 0.23% [0.1–0.4]) and fasting blood glucose values (mean difference, 0.40 mmol/L [0.2–0.7]) in individuals suffering from both T2DM and insomnia compared to the individuals with T2DM only [78]. A cross-sectional study reported the association of insomnia with diabetic retinopathy in 1231 T2DM patients [79]. Moreover, a longitudinal study revealed that insomnia is linked to a higher mortality incidence among individuals with T2DM [80]. Lastly, insomnia has a detrimental impact on quality of life (QoL), impacting all dimensions of QoL assessment measures, particularly in comparison to individuals with T2DM who do not complain of insomnia [81].

#### 3.3.2. Obstructive Sleep Apnea

Another sleep disorder that is highly associated with obesity and T2DM is OSA. OSA is defined by symptoms of total or partial airway failure which are accompanied by a drop in oxygen saturation or awakening from sleep. This disruption can lead to a fragmented, non-restorative sleep [82]. According to a comprehensive review [11], it was found that OSA is more common in individuals with T2DM, with a total OSA occurrence rate varying between 55% and 86%. Males had more severe symptoms and a greater incidence. Obese individuals with T2DM had an 86% occurrence rate of OSA. Because of the common relationship with obesity, establishing an independent relationship between OSA and diabetes is difficult. An additional review demonstrated that intermittent hypoxia and sleep insufficiency have a stimulatory impact on glucose dysregulation and obesity [83]. OSA is a significant determinant of T2DM, with a 49% rise in diabetes risk after controlling variables such as BMI [81]. Furthermore, regardless of BMI, the presence of both OSA and insomnia is related to a greater frequency of cardiovascular events, including diabetes [84].

Despite its prevalence, OSA remains undetected in most individuals with T2DM handled in the healthcare system, with only 18% being discovered [11]. When detected, OSA is related to poor glycemic control with a difference in HbA1c values of 1% between individuals with T2DM depending on OSA severity percentiles [85]. Individuals with T2DM and OSA are more prone to vascular complications, where those patients are more susceptible to developing coronary artery disease and heart failure [86]. In addition to that, they have a greater risk for diabetic retinopathy [87], nephropathy [88], and neuropathy [89]. In contrast to those with either T2DM or OSA alone, individuals having both had a greater risk for CVD death [90]. Lastly, in addition to worsening health outcomes, T2DM patients with OSA also have worse QoL scores across the board [91].

#### 3.3.3. Other Sleep Disorders (NEW)

Other sleep disorders including central sleep apnea, periodic limb movement disorder, and hypersomnolence disorders have also been associated with metabolic dysfunction and increased risk of obesity and diabetes. Excessive daytime sleepiness and abnormal sleep regulation may contribute to impaired glucose metabolism through mechanisms involving autonomic dysregulation, inflammation, and hormonal imbalance [22].

## 4. Circadian Rhythm and Sleep Disturbances in Type 1 Diabetes

**Figure 2:**
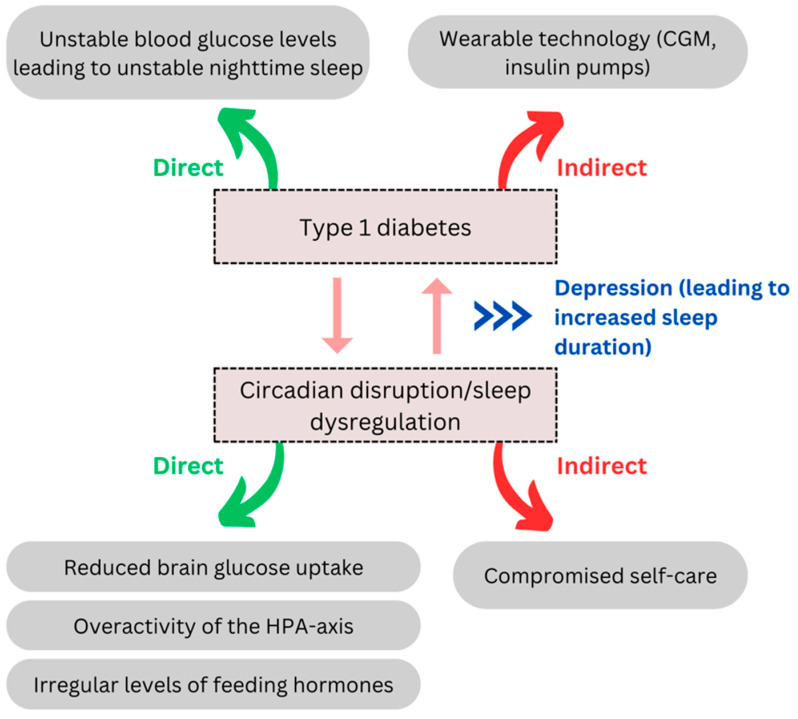
Bidirectional relationship between circadian/sleep disruption and type 1 diabetes. Sleep and circadian disturbances impair glycemic control, while T1DM disrupts sleep via nocturnal glucose fluctuations and management practices, with indirect effects on self-care and lifestyle.

The etiology and pathophysiology of T1DM differ from those of T2DM. Hence the ensuing discussion will primarily focus on a general exploration of the association between sleep dysregulation and T1DM. Several studies have revealed a bidirectional association between sleep disruptions, circadian rhythm disturbances, and T1DM (Figure 2) [5,92].

Firstly, irregular sleep patterns and disrupted circadian rhythms can exert a profound impact on glycemic control through well-defined biological processes including reduced brain glucose uptake, overactivity of the HPA-axis, and irregular levels of feeding hormones such as leptin and ghrelin [5]. Additionally, these disturbances can indirectly influence glycemic control by compromising self-care practices [93].

Insufficient sleep may adversely impact the cognitive functions essential for optimal self-care, such as compromised attentiveness, impaired reasoning, decision-making, and problem-solving abilities [94]. In a study that included adults with T1DM, it was reported that 12% of the participants experienced difficulties in accurately calculating bolus doses due to disturbances during the night, while 33% reported that such disturbances impacted their capacity to make diabetes-related choices [95]. Similarly, a study with adolescents with T1DM found that poor sleep quality was linked to procrastination in glucose monitoring and reduced attentiveness to self-care [96]. Insufficient sleep hours can also contribute to a sedentary lifestyle and the consumption of more undesirable foods such as those heavy in fat and sugar [97]. Furthermore, disrupted sleep can inhibit self-care practices by adversely affecting various aspects of QoL, including physical and mental health, social relationships, and performance in academic or professional settings [95].

On the other way around, diabetes may cause direct disruption of sleep and circadian rhythm when glycemic control during nighttime is unstable leading to symptomatic hypo- and hyperglycemia. Indirect disruption may also occur through the management processes such as the use of wearable technology like continuous glucose monitors (CGMs) and insulin pumps, which can interfere with maintaining uninterrupted sleep due to the need for adjusting blood glucose fluctuations. Additionally, it is worth highlighting the significant association between depression and sleep disturbances in the context of diabetes. For instance, depression may contribute to the links between sleep disruption, the progression of diabetes, and unsatisfactory outcomes. Notably, the strength of these associations diminishes after accounting for the presence of depression, particularly in relation to excessive sleep hours (Figure 2) [61].

## 5. Future Directions

The relationship between circadian rhythm dysregulation, abnormal sleep patterns, and sleep disorders is an area that demands further investigation. Future research may involve a broader range of more thorough studies undertaking modern systematic long-term clinical experiments with theoretical frameworks to explore the effect on specific types of diabetes such as those related to T2DM.

Further investigation into the link between circadian rhythm dysregulation, abnormal sleep patterns, and sleep disorders with the development of diabetes requires larger, longitudinal studies addressing specific research questions in future studies, such as the impact of circadian rhythm dysregulation on different types of diabetes, the interaction with other risk factors like obesity and genetics, and the potential of interventions to prevent or delay diabetes onset and improve glycemic control. These studies should encompass a diverse range of participants and evaluate various factors, including sleep duration, quality, timing, and light exposure. Moreover, identifying the specific mechanisms underlying the contribution of circadian rhythm dysregulation and abnormal sleep patterns to diabetes development necessitates focused research on circadian genes, hormones, and neurotransmitters involved in glucose metabolism.

Furthermore, the development of effective interventions for preventing and handling circadian rhythm disruptions in individuals with diabetes requires further investigation. Promising interventions may include chronotherapy, time-restricted feeding, and cognitive-behavioral therapy for insomnia. At the same time, public health campaigns are essential to raise awareness about the significance of sleep and circadian rhythm health in diabetes prevention and control.

## 6. Limitations

This review has several limitations. First, much of the available evidence is derived from observational and cross-sectional studies, which limits the ability to establish causal relationships between sleep disturbances and diabetes. Second, variability in the assessment of sleep parameters across studies, including both subjective and objective measures, may contribute to heterogeneity in the findings.

Third, potential confounding factors such as obesity, lifestyle behaviors, and comorbid conditions may influence the observed associations. Finally, there is limited evidence regarding the relationship between circadian rhythm disruption and T1DM, highlighting the need for further focused research in this area.

## 7. Conclusions

The development of diabetes is the result of a multifaceted interplay among various factors. Among these factors, compromised sleep patterns and disruptions in the circadian rhythm play a significant role. This relationship is bidirectional, with circadian disruptions potentially contributing to diabetes and vice versa. Moreover, several elements can contribute to circadian disruptions, including daily sleep duration, individual chronotype, SJL, and various sleep disorders.

Understanding this complex interaction and noticing the body’s physiological and biological requirements and regular rhythms can potentially reduce the risk of various metabolic diseases. Healthcare practitioners should prioritize complete sleep assessments and include sleep-related therapies in diabetes prevention and treatment strategies according to the available evidence.

## Figures and Tables

**Table 1:** A presentation of various studies conducted to investigate the effects of long and short sleep durations on the development of T2DM.

Reference	Year	Type of Study	Sample Description	Study Duration	Studies Included in Meta-Analyses	Inference/Major Findings
Lee et al. [66]	2023	Cohort	n = 7407	16 years	-	Short sleep duration (≤5 h) increased the risk for T2DM, particularly among non-obese, male, and <60 of age individuals.
Lu et al. [68]	2021	Meta-analysis	N = 7,373,002	-	17 cohorts	Both short (≤6 h) and long (≥9 h) sleep duration increased the risk of T2DM compared to normal sleep duration (6–9 h).
Mussa et al. [70]	2019	Randomized controlled trial	n = 51	6 months	-	Individualized interventions targeting improved sleep duration demonstrated substantial improvements in body weight, BMI, and HbA1c levels among patients with T2DM.
Jike et al. [69]	2018	Meta-analysis	N = 5,134,036	-	137 prospective cohorts	Long sleep duration is associated with a higher risk of incident DM (RR, 1.26, 95% CI, 1.14–1.37).
Lee et al. [71]	2017	Meta-analysis	N = 29,649	-	8 cross-sectional studies	Short and long sleep durations are associated with increased levels if HbA1c and fasting blood glucose compared to normal sleep duration.
Itani et al. [64]	2017	Meta-analysis	N = 5,172,718	-	108 prospective cohorts	Short sleep durations (<6 h) had an increased risk of 37% for T2DM. Males were more susceptible to the association between fewer sleeping hours and incidence of T2DM compared to females.
Anothaisintawee et al. [67]	2016	Meta-analysis	N = 1,061,555	-	37 cohorts (34 prospective, 3 retrospective)	The risk of developing diabetes due to sleep disturbances such as sleep duration is comparable to that of traditional risk factors.
Chaput et al. [63]	2007	Cross-sectional	N = 740	3 years	-	Sleeping less than 6 h leads to impaired glucose tolerance (IGT). Both inadequate and excessive sleep durations are linked to an increased risk of T2DM and IGT.
Yaggi et al. [59]	2006	Cohort	n = 1709	18 years	-	Short and long sleep durations are proved to increase the risk of T2DM

## Data Availability

No new data were created or analyzed in this study. Data sharing is not applicable to this article.

## Abbreviations

T2DM	Type 2 Diabetes
T1DM	Type 1 Diabetes Mellitus
SNS	Sympathetic Nervous System
FFAs	Free fatty acids
SJL	Social Jetlag
OSA	Obstructive Sleep Apnea
QoL	quality of life
CGMs	continuous glucose monitors
